# The peopling of the last Green Sahara revealed by high-coverage resequencing of trans-Saharan patrilineages

**DOI:** 10.1186/s13059-018-1393-5

**Published:** 2018-02-12

**Authors:** Eugenia D’Atanasio, Beniamino Trombetta, Maria Bonito, Andrea Finocchio, Genny Di Vito, Mara Seghizzi, Rita Romano, Gianluca Russo, Giacomo Maria Paganotti, Elizabeth Watson, Alfredo Coppa, Paolo Anagnostou, Jean-Michel Dugoujon, Pedro Moral, Daniele Sellitto, Andrea Novelletto, Fulvio Cruciani

**Affiliations:** 1 Dipartimento di Biologia e Biotecnologie “C. Darwin”, Sapienza Università di Roma, Rome, Italy; 2 Istituto Pasteur-Fondazione Cenci Bolognetti, Sapienza Università di Roma, Rome, Italy; 3 Dipartimento di Biologia, Università di Roma “Tor Vergata”, Rome, Italy; 4 Dipartimento di Sanità Pubblica e Malattie Infettive, Sapienza Università di Roma, Rome, Italy; 5 Botswana-University of Pennsylvania Partnership, Gaborone, Botswana; 6 Perelman School of Medicine, University of Pennsylvania, Philadelphia, PA USA; 7 The Swedish Museum of Natural History, Stockholm, Sweden; 8 Dipartimento di Biologia Ambientale, Sapienza Università di Roma, Rome, Italy; 9 Istituto Italiano di Antropologia, Rome, Italy; 10 Centre National de la Recherche Scientifique (CNRS), Université Toulouse-3–Paul-Sabatier, Toulouse, France; 11 Department of Animal Biology-Anthropology, Biodiversity Research Institute, University of Barcelona, Barcelona, Spain; 12 Istituto di Biologia e Patologia Molecolari, CNR, Rome, Italy

**Keywords:** MSY, Target next generation sequencing, Green Sahara, Trans-Saharan haplogroups

## Abstract

**Background:**

Little is known about the peopling of the Sahara during the Holocene climatic optimum, when the desert was replaced by a fertile environment.

**Results:**

In order to investigate the role of the last Green Sahara in the peopling of Africa, we deep-sequence the whole non-repetitive portion of the Y chromosome in 104 males selected as representative of haplogroups which are currently found to the north and to the south of the Sahara. We identify 5,966 mutations, from which we extract 142 informative markers then genotyped in about 8,000 subjects from 145 African, Eurasian and African American populations. We find that the coalescence age of the trans-Saharan haplogroups dates back to the last Green Sahara, while most northern African or sub-Saharan clades expanded locally in the subsequent arid phase.

**Conclusions:**

Our findings suggest that the Green Sahara promoted human movements and demographic expansions, possibly linked to the adoption of pastoralism. Comparing our results with previously reported genome-wide data, we also find evidence for a sex-biased sub-Saharan contribution to northern Africans, suggesting that historical events such as the trans-Saharan slave trade mainly contributed to the mtDNA and autosomal gene pool, whereas the northern African paternal gene pool was mainly shaped by more ancient events.

**Electronic supplementary material:**

The online version of this article (10.1186/s13059-018-1393-5) contains supplementary material, which is available to authorized users.

## Background

The Sahara desert is the widest hot desert on Earth and covers about one-third of the African continent, from the Atlantic coast to the Red Sea. Over the past millennia, the Sahara underwent strong climatic fluctuations, alternating arid and humid phases. During the humid periods, referred to as “Green Sahara” or “African humid periods”, the landscape was characterised by the presence of savannah, forests and an extensive system of rivers and lakes [1–3]. A large amount of paleoecological and paleoanthropological evidence indicates that the fertile environment probably enabled the occupation of the Saharan area by fauna and hominins since the Miocene [3–5].

The most recent Green Sahara period occurred in the Holocene, in a time frame from about 12 thousand of years ago (kya) to about 5 kya. This phase has been denominated the “Holocene climatic optimum” and is the most well-documented past climatic change [2, 6]. Human settlement across the Sahara in this period is testified by archaeological evidence, such as rock engravings, lithic and bone tools and pottery [7].

After the African humid period, the climatic conditions became rapidly hyper-arid and the Green Sahara was replaced by the desert, which acted as a strong geographic barrier against human movements between northern and sub-Saharan Africa.

A consequence of this is that there is a strong differentiation in the Y chromosome haplogroup composition between the northern and sub-Saharan regions of the African continent. In the northern area, the predominant Y lineages are J-M267 and E-M81, with the former being linked to the Neolithic expansion in the Near East and the latter reaching frequencies as high as 80 % in some north-western populations as a consequence of a very recent local demographic expansion [8–10]. On the contrary, sub-Saharan Africa is characterised by a completely different genetic landscape, with lineages within E-M2 and haplogroup B comprising most of the Y chromosomes. In most regions of sub-Saharan Africa, the observed haplogroup distribution has been linked to the recent (~ 3 kya) demic diffusion of Bantu agriculturalists, which brought E-M2 sub-clades from central Africa to the East and to the South [11–17]. On the contrary, the sub-Saharan distribution of B-M150 seems to have more ancient origins, since its internal lineages are present in both Bantu farmers and non-Bantu hunter-gatherers and coalesce long before the Bantu expansion [18–20].

In spite of their genetic differentiation, however, northern and sub-Saharan Africa share at least four patrilineages at different frequencies, namely A3-M13, E-M2, E-M78 and R-V88.

A3-M13 is typical of eastern Africa, where it is found with a frequency as high as 40 % and is prevalent in the Nilo-Saharan populations, in particular among Nilotic pastoralists [14, 18, 21]. A3-M13 chromosomes have also been observed in central and northern Africa, at frequencies ranging from 1 to 7 % [12, 18, 22, 23]. Outside Africa, this haplogroup has been found at very low frequency in both the Middle East and Sardinia [23–30].

As described above, E-M2 is a sub-Saharan clade which has been often associated with the Bantu expansion. However, E-M2 chromosomes have also been found at low frequencies (2–10 %) in northern Africa [8, 9, 22, 23, 31, 32].

E-M78 is a widespread lineage, with significant frequencies in Africa, Europe and the Middle East [33, 34]. Within the African continent, three E-M78 sub-clades (E-V22, E-V12 and E-V264) show different frequencies in different regions. E-V22 is mainly an eastern African sub-haplogroup, with frequencies of more than 80 % in the Saho population from Eritrea, but it has also been reported in Egypt and Morocco [34–36]. E-V12 is relatively frequent in northern and eastern Africa, but it has also been reported outside Africa at lower frequencies [33–35]. The vast majority of the eastern African E-V12 chromosomes belong to the internal clade E-V32, which has also been observed in northern and central Africa at very low frequencies [12, 33–35]. E-V264 is subdivided into two sub-clades: E-V65, common in northern Africa; and E-V259, which includes few central African chromosomes [33–35].

R-V88 has been observed at high frequencies in the central Sahel (northern Cameroon, northern Nigeria, Chad and Niger) and it has also been reported at low frequencies in northwestern Africa [37]. Outside the African continent, two rare R-V88 sub-lineages (R-M18 and R-V35) have been observed in Near East and southern Europe (particularly in Sardinia) [30, 37–39]. Because of its ethno-geographic distribution in the central Sahel, R-V88 has been linked to the spread of the Chadic branch of the Afroasiatic linguistic family [37, 40].

From a genetic point of view, the use of variability in the present-day male-specific portion of the human Y chromosome (MSY) to infer past population dynamics across the Sahara is complicated by two major factors: 1) the onset of the hyper-arid conditions caused the depopulation of the Sahara; 2) the regions immediately northward and southward of the Sahara have experienced extensive demographic expansions after the African humid period, which have led to the increase in frequency of different Y haplogroups, partially concealing the pre-existing genetic composition [32, 41].

In this context, rare Y lineages with a relic geographic distribution can be highly informative regarding human migrations across the Sahara. Thus, considering their frequency distribution, the four trans-Saharan lineages A3-M13, E-M2, E-M78 and R-V88 could represent the remains of the Saharan MSY genetic landscape before the desertification, contrary to the usual interpretation involving recent gene flow events such as the trans-Saharan Arab slave trade [42–44].

In order to investigate the role of the last Green Sahara in the peopling of Africa, we performed targeted next generation sequencing (NGS) of ~ 3.3 Mb of 104 Y chromosomes mostly belonging to these four lineages. We also analysed the geographic distribution of 142 informative single nucleotide polymorphisms (SNPs) by genotyping about 8000 male subjects from 145 world-wide populations (including 17 populations from literature), with a particular focus on the African ethnic groups. Our findings were consistent with the hypothesis that the Green Sahara allowed extensive human movements, excluding recent historical events, such as the Arab slave trade, as a major determinant of the male gene pool of present-day northern African populations.

## Results

### Phylogenetic tree and the four trans-Saharan clades

**Fig. 1:**
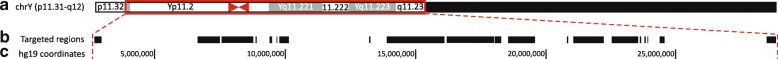
Regions of the MSY selected for the target next generation sequencing. **a** The human Y chromosome. **b** Targeted blocks of the X-degenerate portion of the MSY analysed in this study (the exact coordinates on the Y chromosome are reported in Additional file 1: Table S6 and a description of the selection criteria is reported in the “Methods” section). **c** Y chromosome ruler calibrated on the February 2009 (GRCh37/hg19) assembly

For the phylogenetic tree reconstruction and time estimates, we used 150 Y chromosomes. The samples were analysed for ~ 3.3 Mb of the X-degenerated portion of the MSY (Fig. 1) and belonged to different datasets: 104 Y chromosomes from our lab collection (77 of them belonging to the four trans-Saharan haplogroups) and 46 publicly available high-coverage sequences, including four precisely radiocarbon-dated ancient specimens as calibration points [45–49] (Additional file 1: Table S1).

**Fig. 2:**
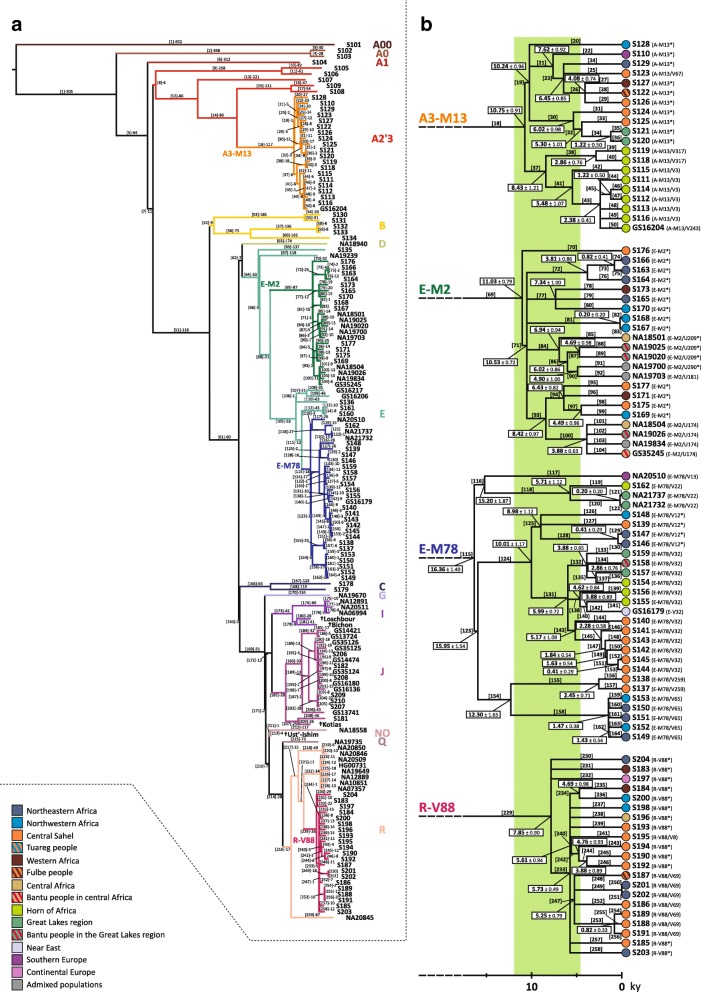
Maximum parsimony Y chromosome tree and dating of the four trans-Saharan haplogroups. **a** Phylogenetic relations among the 150 samples analysed here. Each haplogroup is labelled in a different colour. The four Y sequences from ancient samples are marked by the *dagger symbol*. **b** Phylogenetic tree of the four trans-Saharan haplogroups, aligned to the timeline (at the *bottom*). At the tip of each lineage, the ethno-geographic affiliation of the corresponding sample is represented by a *circle*, coloured according to the legend (*bottom left*). The last Green Sahara period is highlighted by a *green belt* in the background

In the set of 104 samples from our lab collection, we identified 5966 SNPs. Interestingly, 3044 variants (51 %) out of the 5966 were not reported in previous studies [30, 48, 50, 51] and this figure is significantly greater than that reported by Hallast et al. [50] (51 *vs* 36.6 %, Chi-squared test: *p* < 2.2 × 10^−16^), despite the fact the experimental approaches were similar (target sequencing) and the number of sequenced samples by Hallast and collegues [50] was about four times higher (Additional file 2: Figure S1). After the inclusion of the 46 samples from the literature [45–49], the total number of variants increased to 7544 (Additional file 1: Table S2). We used all 7544 SNPs in the whole set of 150 subjects to reconstruct a maximum parsimony tree (Fig. 2a), which was found to be coherent with the recently published world-wide Y phylogenies [48, 51].

By calibration with the four archeologically dated specimens, we obtained a mutation rate of 0.735 × 10^−9^/site/year, which is consistent with previously published estimates [47, 51, 52] and which was used to obtain an accurate estimate of the coalescence age of the tree nodes, with a particular focus on the four trans-Saharan clades. We obtained the time estimates using two different approaches: Rho statistics (Table 1) and the BEAST method. We performed two different BEAST runs, under a strict or a relaxed clock, respectively (Additional file 1: Table S3). The obtained point values were found to be highly concordant (Pearson test, R^2^ > 0.99; *p* < 2.2 × 10^−16^), as previously observed [19] (Additional file 2: Figure S2). For this reason, hereafter we only report and discuss the time estimates based on the Rho statistics (Fig. 2b).

**Table 1:** Time estimates for the nodes of the phylogenetic tree

Haplogroup	Node	Rho	SD Rho	Time (kya)	SD time (kya)
-	Root	628.78	20.46	256.54	8.35
A0'T	1	420.48	14.81	171.55	6.04
A0	2	29.00	3.81	11.83	1.55
A1'T	5	359.57	12.71	146.71	5.19
A2'T	7	348.91	12.36	142.35	5.94
A2'3	8	310.72	14.64	126.77	5.97
A2-PN3	9	71.50	5.98	29.17	2.44
A3-M32	12	224.09	12.89	91.43	5.26
A3-M144	14	144.18	10.06	58.83	4.10
A3-M51	15	50.50	5.02	20.60	2.05
A3-M13	18	26.35	2.23	10.75	0.91
A3-M13	19	25.09	2.36	10.24	0.96
A3-M13	21	18.67	2.25	7.62	0.92
A3-M13	23	15.80	2.09	6.45	0.85
A3-M13	26	10.00	1.83	4.08	0.74
A3-M13	30	14.75	2.41	6.02	0.98
A3-M13	32	13.00	2.47	5.30	1.01
A3-M13	34	3.00	1.22	1.22	0.50
A3-M13	37	20.67	2.97	8.43	1.21
A3-M13	38	7.00	1.87	2.86	0.76
A3-M13	41	13.43	2.62	5.48	1.07
A3-M13	43	5.83	1.01	2.38	0.41
A3-M13	45	3.00	1.22	1.22	0.50
B'T	51	238.79	9.90	97.42	4.04
B	52	222.00	9.39	90.58	3.83
B-M236	53	32.00	4.00	13.06	1.63
B-M182	56	150.00	8.96	61.20	3.65
B-M150	57	8.00	2.00	3.26	0.82
D'T	61	180.13	6.91	73.49	2.82
DE-YAP	62	175.36	9.70	71.55	3.96
E-M40	64	142.39	8.03	58.09	3.28
E-P110	66	139.48	7.98	56.91	3.26
E-P2	68	118.87	6.71	48.50	2.74
E-M2	69	27.05	1.95	11.03	0.79
E-M2	71	25.81	1.76	10.53	0.72
E-M2	72	9.33	2.11	3.81	0.86
E-M2	73	2.00	1.00	0.82	0.41
E-M2	77	18.00	2.45	7.34	1.00
E-M2	81	0.50	0.50	0.20	0.20
E-M2	84	17.00	2.31	6.94	0.94
E-M2	86	14.75	2.11	6.02	0.86
E-M2	87	11.50	2.40	4.69	0.98
E-M2	90	12.00	2.45	4.90	1.00
E-M2	93	20.63	2.37	8.42	0.97
E-M2	94	15.75	2.02	6.43	0.82
E-M2	97	11.00	2.35	4.49	0.96
E-M2	100	9.50	1.54	3.88	0.63
E-M35	105	69.10	5.63	28.19	2.30
E-M35	106	62.00	5.03	25.30	2.05
E-M35	107	40.50	4.50	16.52	1.84
E-V68	111	57.70	5.11	23.54	2.08
E-V68	112	9.00	2.12	3.67	0.87
E-M78	115	40.11	3.42	16.36	1.40
E-M78	116	37.25	4.59	15.20	1.87
E-M78	118	14.00	2.75	5.71	1.12
E-M78	120	0.50	0.50	0.20	0.20
E-M78	123	39.08	3.78	15.95	1.54
E-M78	124	24.53	2.86	10.01	1.17
E-M78	125	22.00	2.74	8.98	1.12
E-M78	128	1.00	0.71	0.41	0.29
E-M78	131	14.69	1.76	5.99	0.72
E-M78	132	9.50	1.58	3.88	0.65
E-M78	135	7.00	1.87	2.86	0.76
E-M78	138	11.33	2.05	4.62	0.84
E-M78	140	9.50	2.18	3.88	0.89
E-M78	143	12.67	2.68	5.17	1.09
E-M78	145	5.60	1.41	2.28	0.58
E-M78	147	4.50	1.32	1.84	0.54
E-M78	149	4.00	1.33	1.63	0.54
E-M78	151	1.00	0.71	0.41	0.29
E-M78	154	30.14	4.04	12.30	1.65
E-M78	155	6.00	1.73	2.45	0.71
E-M78	158	3.60	0.94	1.47	0.38
E-M78	162	3.50	1.32	1.43	0.54
C'T	165	175.74	9.32	71.70	3.80
C	166	118.00	7.68	48.14	3.13
G'T	169	124.54	6.52	50.81	2.66
I'T	171	122.35	6.49	49.92	2.65
IJ	172	106.89	7.37	43.61	3.01
I	173	12.33	2.13	5.03	0.87
I-M253	176	11.50	2.40	4.69	0.98
J	182	76.38	6.68	31.16	2.73
J-M267	183	43.07	4.19	17.57	1.71
J-M267	184	11.00	2.35	4.49	0.96
J-M267	187	42.08	4.64	17.17	1.89
J-M267	188	28.83	3.45	11.76	1.41
J-M267	189	17.78	2.44	7.25	1.00
J-M267	191	12.75	1.51	5.20	0.62
J-M267	193	11.00	1.27	4.49	0.52
J-M267	198	9.00	2.12	3.67	0.87
J-M267	202	5.00	1.29	2.04	0.53
K	210	116.81	8.74	47.66	3.57
Q'R	214	88.77	7.30	36.22	2.98
R	216	72.30	6.31	29.50	2.57
R-M173	217	56.79	5.25	23.17	2.14
R-M420	218	8.00	2.00	3.26	0.82
R-V1501	221	45.78	4.52	18.68	1.85
R-P312/U152	222	13.67	1.51	5.58	0.62
R-V88	229	19.24	2.20	7.85	0.90
R-V88	233	14.06	1.19	5.73	0.49
R-V88	234	11.50	2.40	4.69	0.98
R-V88	240	13.75	2.30	5.61	0.94
R-V88	242	11.67	2.29	4.76	0.93
R-V88	244	9.50	2.18	3.88	0.89
R-V88	247	12.86	1.95	5.25	0.79
R-V88	253	2.00	0.82	0.82	0.33

For each node, we report Haplogroup, node number (Fig. 2), Rho statistics, time estimate in thousands of years ago (kya) and their standard deviation (SD)

A3-M13 phylogeny is characterised by a first bifurcation separating branches 19 and 37 about 10.75 kya. Interestingly, branch 19 has a widespread distribution, harbouring lineages from within and outside the African continent, and is dated to 10.24 kya, suggesting a role of the humid period in the diffusion of this clade. On the contrary, branch 37 only includes samples from the Horn of Africa (Ethiopia, Eritrea, Djibouti and Somalia) and is dated to 8.43 kya.

The topology of E-M2 is characterised by a main multifurcation (downstream to branch 71), dating back to the beginning of the last Green Sahara (10.53 kya) and including all the deep-sequenced samples except one (branch 70), consistent with the tree reported in phase 3 of the 1000 Genomes Project [51]. However, we found 11 subclades (branches 72, 73, 74, 75, 76, 79, 81, 82, 95, 98 and 99) which share no markers with the 262 E-M2 chromosomes analysed by Poznik and collegues [51]. It is worth noting that branches 72 and 81 are two deep sister lineages within the E-M2 main multifurcation (Fig. 2) and both of them include chromosomes from northern Africa. Similarly, the other terminal lineages absent in the 1000 Genomes Project’s tree are mainly represented by samples from northern Africa or, to a lesser extent, from the northernmost regions of sub-Saharan Africa (i.e. the central Sahel) (Fig. 2b).

The phylogenetic structure of E-M78 has been resolved in a recent study [35]; however, we obtained further information about the relationships within the E-V12 sub-clade. The former E-V12* chromosomes form a monophyletic cluster (branch 125), dated to 8.98 kya and sister to E-V32 (branch 131), which in turn is further subdivided into three sister clades (branches 132, 138 and 143). While branches 132 and 138 have been found in eastern Africa, where E-V32 is more frequent, branch 143 only includes samples from central Sahel (Fig. 2b).

Finally, the R-V88 lineages date back to 7.85 kya and its main internal branch (branch 233) forms a “star-like” topology (“Star-like” index = 0.55), suggestive of a demographic expansion. More specifically, 18 out of the 21 sequenced chromosomes belong to branch 233, which includes eight sister clades, five of which are represented by a single subject. The coalescence age of this sub-branch dates back to 5.73 kya, during the last Green Sahara period. Interestingly, the subjects included in the “star-like” structure come from northern Africa or central Sahel, tracing a trans-Saharan axis. It is worth noting that even the three lineages outside the main multifurcation (branches 230, 231 and 232) are sister lineages without any nested sub-structure. The peculiar topology of the R-V88 sequenced samples suggests that the diffusion of this haplogroup was quite rapid and possibly triggered by the Saharan favourable climate (Fig. 2b).

In general, our NGS results and time estimates show that the large majority of the lineages shared by northern Africans and sub-Saharan Africans coalesced during the last Green Sahara period. Conversely, after 5 kya, we mainly found lineages restricted to either northern or sub-Saharan regions, with few exceptions (Fig. 2b).

### Population analysis of the four trans-Saharan clades

**Fig. 3:**
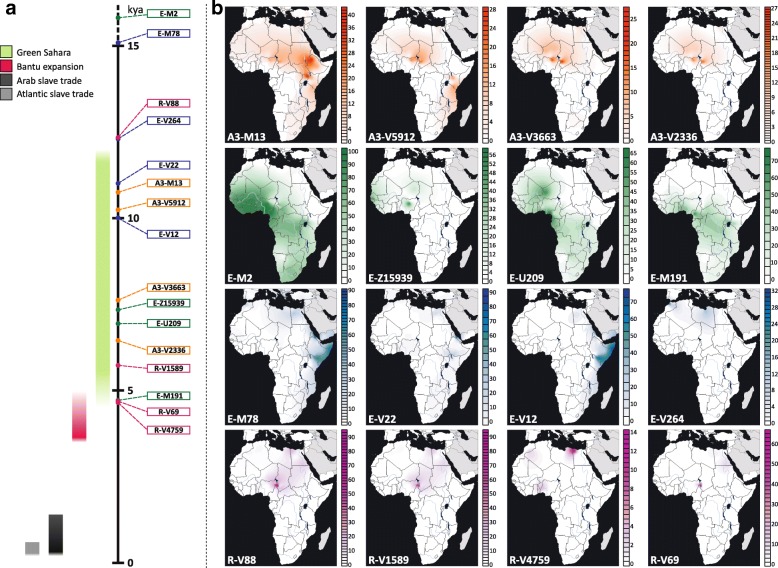
Time estimates and frequency maps of the four trans-Saharan haplogroups and major sub-clades. **a** Time estimates of the four trans-Saharan clades and their main internal lineages. To the *left* of the timeline, the time windows of the main climatic/historical African events are reported in different colours (legend in the *upper left*). **b** Frequency maps of the main trans-Saharan clades and sub-clades. For each map, the relative frequencies (percentages) are reported to the right

**Fig. 4:**
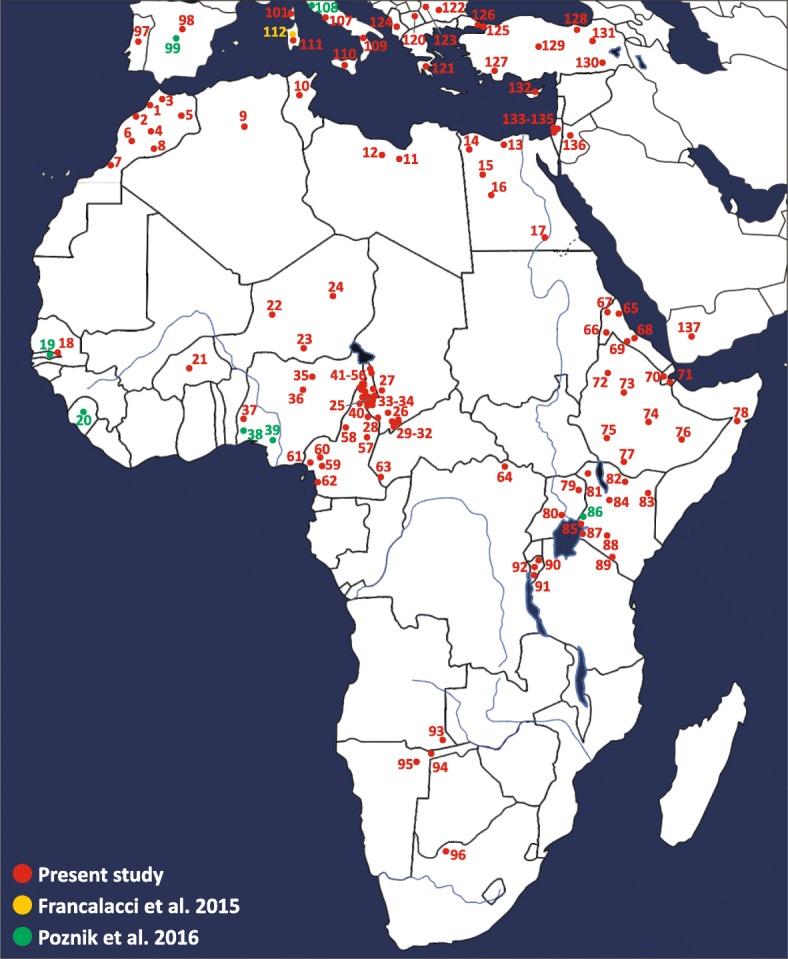
Map of the populations analysed. Geographic positions of the populations from Africa, southern Europe and Near East are shown. For population labels refer to Additional file 1: Table S5

In order to gain more information about the ethno-geographic distribution of the four trans-Saharan haplogroups (Fig. 3), we selected 142 informative markers (Additional file 1: Table S4) belonging to these lineages and analysed them in a wider sample composed of 7955 males from 145 worldwide populations (128 from our lab collection and 17 from the literature) (Fig. 4) [51, 53] (Additional file 1: Table S5). It is worth noting that 96 ethnic groups come from different African regions, allowing us to obtain a detailed picture of the genetic variability of the four haplogroups across the Sahara (Figs. 3 and 4).

We also included eight admixed populations from America [51], whose genetic variation has been shaped by the trans-Atlantic slave trade (XV–XIX centuries), to be used as a “positive control” to investigate the effects of other recent historical events, such as the Arab slave trade (VII–XIX centuries) which involved the forced movement of millions of sub-Saharan Africans toward northern Africa [54] (see “Discussion”).

The genotyping results for A3-M13 confirmed its very high geographic differentiation, with most lineages restricted to one geographic area. There are few exceptions to this general pattern, i.e. A3-V2742*, A3-V2816* and A3-V3800, which were found in two different regions, usually belonging to the same geographical macro-area (Additional file 2: Figure S3). While A3-V1018 is restricted to the Horn of Africa, its sister clade, A3-V5912, is more widespread, arriving as far as southern Europe (more specifically, Sardinia) (Additional file 1: Table S5). Most of the Mediterranean lineages coalesced with sub-Saharan clades in a time window between 10.24 and 6.45 kya (where the upper and lower limit are the coalescence ages of A3-V5912 and A3-V2336, respectively) (Fig. 3b), during the last humid phase of the Sahara (12–5 kya). After this period, the lineages are restricted to sub-Saharan Africa or northern Africa. It is worth noting that A3-V4735 has been found both in central Sahel and in the Great Lakes region (Kenya and Uganda) in eastern Africa, suggesting a movement along the Sahelian belt starting during the final period of the last Green Sahara (6.02–5.30 kya).

It is known that the geographic distribution of E-M2 in sub-Saharan Africa has been heavily influenced by the recent (< 3 kya) Bantu expansion [11–17] and this is mirrored by the high frequencies of several E-M2 sub-clades among the Bantu people, in particular E-U290 and E-U174 (Additional file 1: Table S5 and Additional file 2: Figure S4). However, we found clues as to the role of the last Green Sahara considering the phylogeography of the E-M2 sub-clades in northern Africa. The coalescence age of the lineages harbouring northern and sub-Saharan chromosomes predates the onset of the arid conditions, falling between 11.03 kya (coalescence age of E-Page66) and 4.49 kya (the time estimate of the most recent clade harbouring a relevant proportion of northern African samples, i.e. E-V5280), during the last Green Sahara. After this time frame, we observed clades restricted to the north or to the south of the Sahara. In this context, although the large majority of the geographically restricted lineages come from sub-Saharan regions, we also found two northern African-specific clades, namely E-V5001 and E-V4990. E-V5001 has only been found in Egypt, is one of the sister clades within the E-M4727 multifurcation and coalesced at 3.88 kya. E-V4990 is a Moroccan clade dated to < 4.49 kya (the time estimate of the upstream node). Interestingly, it is the terminal branch of a nested topology, which divides western Africa from Morocco. We found a relevant proportion (~ 22 %) of African-American subjects belonging to the E-M2 haplogroup (Additional file 1: Table S5). These groups have been heavily influenced by the Atlantic slave trade, which took place between the XV and XIX centuries and of which the source populations were mainly sub-Saharan people. Consistent with the autosomal data [55], these subjects have been found to be very similar to the source African populations in their E-M2 sub-haplogroup composition (Additional file 2: Figure S4).

The distribution and age estimates of different E-M78 sub-haplogroups show a strong parallelism. Excluding the E-V13 subclade, which has been linked to the Neolithic transition in the Near East [34], all the other three major E-M78 lineages (E-V264, E-V22 and E-V12) include a Mediterranean clade (harbouring northern African, near-eastern and southern European samples) and a sub-Saharan clade (Fig. 3b; Additional file 2: Figure S5). The age estimates of the nodes joining the lineages from these two macro-areas are quite concordant (12.30 kya for E-V264, 11.01 kya for E-V22 and 10.01 kya for E-V12) and correspond to the beginning of the humid phase in the eastern Sahara, where E-M78 probably originated [34, 35]. After the end of the last Green Sahara (~ 5 kya), the differentiation is sharp, with no lineages including both Mediterranean and sub-Saharan subjects. The sub-Saharan clades E-V264/V259 and E-V22/V3262 are restricted to central Sahel and eastern Africa (mainly the Horn of Africa), respectively, whereas E-V12/V32 is very frequent in eastern Africa but it also includes a central Sahelian clade, suggesting a Sahelian movement between 5.99 and 5.17 kya.

The genotyping of R-V88 internal markers disclosed the phylogenetic relationships of two rare European sub-clades (R-M18 and R-V35) with respect to African-specific clades (Additional file 2: Figure S6). The presence of two nested R-V88 basal European clades can be related to the high frequencies of R-V88 internal lineages in the central Sahel assuming a movement from Europe toward the central Sahel across northern Africa. In turn, considering the trans-Saharan distribution and the “star-like” topology of the sub-clade R-V1589 (branch 233), it is likely that this lineage rapidly expanded in the lake Chad area between 5.73 and 5.25 kya and moved backward to northeastern Africa across the Saharan region (Fig. 3b; Additional file 2: Figure S6). The large majority of R-V1589 internal lineages harbours both northern and central Sahelian subjects, with the exception of R-V4759 and R-V5781, which are mainly restricted to northern Africa and central Sahel, respectively (Additional file 1: Table S5). The presence of a precisely dated and geographically restricted clade (R-V4759 in northern Africa; Additional file 1: Table S5 and Additional file 2: Figure S6) allowed us to define its coalescence age (4.69 kya) as the lower limit for the backward R-V88 trans-Saharan movement.

### Beyond the last Green Sahara

Although the focus of the present study was to understand the African population dynamics linked to the last Green Sahara period, we also found evidence of other movements within and outside Africa involving different ethnic groups and that occurred before or after the Holocene climatic optimum.

The Sahelian belt spans from the Atlantic Ocean to the Red Sea, immediately south of the Sahara. Its climate and ecology are intermediate between desert (typical of the Sahara to the north) and the tropical savannah (typical of the regions to the south). In this area, several languages belonging to three of the four African linguistic families (i.e. Afro-asiatic, Nilo-Saharan and Niger-Congo) are spoken, confirming that the Sahel has been an important crossroad in the African continent. We found evidence of Sahelian movements in at least three haplogroups: A3-M13/V4735, E-M2/Z15939 and E-M78/V32 (Additional file 2: Figures S2–S4). A3-M13/V4735 and E-M78/V32 seem to have been involved in human movements that occurred in the same time window (~ 6–5 kya) along the same bi-directional Sahelian axis from lake Chad to eastern Africa. A3-M13/V4735 probably originated somewhere in the central Sahel between 10.24 and 6.02 kya and possibly arrived in eastern Africa after 6.02 kya. This clade is significantly related to the Nilo-Saharan speaking groups (Mann–Whitney test, *p* = 2.82 × 10^−4^), refining previous hypotheses about the association between A3-M13 and the Nilo-Saharan spread from central Sahel to eastern Africa [14, 18]. In the same period, we found evidence of a movement along the same Sahelian axis involving the internal lineages of E-M78/V32. This haplogroup probably differentiated in eastern Africa 5.99 kya, and we observed a shift in its geographic distribution towards the central Sahel, where it arrived not later than 5.17 kya. Interestingly, all the central sahelian E-V32 chromosomes belong to the internal clade E-V32/V6873, which is almost exclusively found among the Nilo-Saharans (Mann–Whitney test, *p* = 0.01). These findings suggest that the Nilo-Saharan spread along the Sahelian belt was probably a complex event, involving different clades and different movements from the lake Chad basin to eastern Africa and back. Haplogroup E-M2/Z15939, whose coalescence age (~ 7 kya) falls within the last Green Sahara period, seems to have been involved in another Sahelian movement, being present at high frequencies among different Fulbe groups. Interestingly, the geographic distribution of this clade (Fig. 3b) perfectly traces the Fulbe migration from western Africa, where this haplogroup is also common in other ethnic groups, to central Sahel, where the same haplogroup is only found among Fulbe populations.

Outside Africa, both A3-M13 and R-V88 harbour sub-lineages geographically restricted to the island of Sardinia and both seem to indicate ancient trans-Mediterranean contacts. The phylogeography of A3-M13 suggests that the direction of the movement was from Africa to Sardinia, while R-V88 topology indicates a Europe-to-Africa migration. Indeed, our data suggest a European origin of R-V88 about 12.3 kya, considering both the presence of two Sardinian R-V88 basal clades (R-M18 and R-V35) and that the V88 marker arose in the R-M343 background, which in turn includes Near-Eastern/European lineages [52]. It is worth noting that the arrival of R-V88 in the Sahara seems to have occurred between 8.67 and 7.85 kya (considering as an upper limit the time estimates of the last node including a European-specific lineage, while the lower limit is the coalescence age of all the African-specific lineages), refining the time frame of the trans-Saharan migration proposed in previous studies [37, 56]. The route of R-V88 toward the lake Chad basin probably passed through northeastern Africa rather than Arabia, considering the absence of R-V88 in the Horn of Africa. Interestingly, both A3-M13 and R-V88 European sub-clades coalesced in ancient times (> 7.62 kya for A3-M13/V2742 and between 12.34 and 8.67 kya for R-V88/M18 and R-V88/V35) (Additional file 2: Figures S2 and S5). So it is possible that both clades were widespread in southern Europe, where they have been replaced by the Y haplogroups brought by the following recurrent migration waves from Asia [57].

## Discussion

### Role of the Green Sahara in the distribution of the four haplogroups

**Fig. 5:**
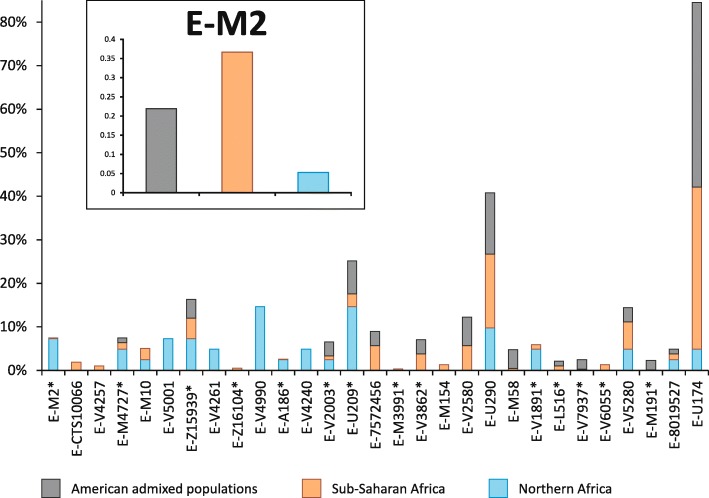
Relative proportions of American admixed, sub-Saharan or northern African Y chromosomes belonging to the E-M2 sub-clades. Data from the nomadic populations (Tuareg and Fulbe) and from seven lineages with an absolute frequency equal to 1 were not used for the generation of this graph. Compared to the macroregion sub-division reported in Additional file 1: Table S5, we collapsed “Northeastern Africa” and “Northwestern Africa” macroregions into “Northern Africa”, while the “Sub-Saharan Africa” group includes “Central Sahel”, “Western Africa”, “Central Africa”, “Great Lakes region”, “Horn of Africa”, “Southern Africa” and all the Bantu groups in these regions. In the *inset*, we report the relative frequencies of the whole E-M2 haplogroup in the same macroregions

The large majority of nodes joining northern and sub-Saharan patrilineages date back to the Green Sahara period. On the contrary, most clades geographically restricted to one of these two macro-regions coalesced after 5 kya. Usually, the presence of a sub-Saharan genetic component in northern Africa is put down to the Arab slave trade (VII–XIX centuries) from the sub-Saharan regions towards the markets located along the Mediterranean coast [42–44]. If this was the case, we should observe no significant differences in the sub-Saharan component of Y haplogroups between the African American and northern African populations, since both the Atlantic and the Arab slave trade are recent events, which involved the same source geographic area (Fig. 3a). However, considering the distribution of E-M2 sub-lineages in the American admixed, northern African and sub-Saharan populations (Fig. 5), we found a significant correlation between admixed and sub-Saharan groups (Spearman’s Rho = 0.687, *p* = 3.76 × 10^−6^) consistent with the genome-wide data [55, 58], while northern Africans and sub-Saharan people were not correlated (Spearman’s Rho = 0.07, *p* = 0.68). Consistent with these findings, also northern Africans and American admixed people were found not to be correlated (Spearman’s Rho = 0.22, *p* = 0.19).

The same pattern was also observed when only the western-central Sahelian groups of sub-Saharan Africa were considered (admixed *vs.* western-central Sahel, Spearman’s Rho = 0.509, *p* = 1.51 × 10^−3^; northern Africa *vs* western-central Sahel, Spearman’s Rho = 0.218, *p* = 0.2). These data suggest that the presence in northern Africa of sub-Saharan patrilineages was not due to recent contacts but probably occurred in more ancient times, possibly during the Green Sahara period considering the coalescence ages of the clades. Our findings seem to be at odds with genome-wide studies [42, 43, 59, 60] reporting a recent relevant sub-Saharan genetic component in modern northern African populations, mainly attributed to the Arab slave trade. This apparent discrepancy between inferences based on Y chromosomal and autosomal data could be the consequence of a sex-biased sub-Saharan contribution to the northern African gene pool that occurred in historical times. Indeed, it is known that the trans-Saharan Arab slave trade involved twice as many servile women as men (almost the reverse of the Atlantic slave trade ratio). Moreover, few male slaves left descendants, whereas female slaves were imported in northern Africa as household servants and as concubines and their offspring were born free, thus contributing to the local gene pool [54, 61]. Thus, we suggest that the Arab slave trade mainly contributed to the mtDNA and autosomal gene pool of present-day northern Africans, whereas the paternal gene pool was mainly shaped by more ancient events. This hypothesis is in line with genome-wide data obtained from three ancient Egyptian mummies (dated between ~ 2.5 and 2 kya) showing a not negligible ancient sub-Saharan component (~ 6–10 %) [44].

Considering the data for all the four trans-Saharan haplogroups reported here, we can try to paint a comprehensive picture of the events during the last African humid period. The first occupation of the Sahara may have occurred from both northern and southern regions, following the spread of the fertile environment and according with the two-way occupation of the Green Sahara proposed on the basis of paleoanthropological evidence [2]. The topology and geographic distribution (Additional file 2: Figures S3 and S4) of both A3-M13 and E-M2 suggest that these lineages were brought to the Sahara from the southern regions, while E-M78 and R-V88 seem to have followed the opposite route.

The fertile environment established in the Green Sahara probably promoted demographic expansions and rapid dispersals of the human groups, as suggested by the great homogeneity in the material culture of the early Holocene Saharan populations [62]. Our data for all the four trans-Saharan haplogroups are consistent with this scenario, since we found several multifurcated topologies, which can be considered as phylogenetic footprints of demographic expansions. The multifurcated structure of the E-M2 is suggestive of a first demographic expansion, which occurred about 10.5 kya, at the beginning of the last Green Sahara (Fig. 2; Additional file 2: Figure S4). After this initial expansion, we found that most of the trans-Saharan lineages within A3-M13, E-M2 and R-V88 radiated in a narrow time interval at 8–7 kya, suggestive of population expansions that may have occurred in the same time (Fig. 2; Additional file 2: Figures S3, S4 and S6). Interestingly, during roughly the same period, the Saharan populations adopted pastoralism, probably as an adaptive strategy against a short arid period [1, 62, 63]. So, the exploitation of pastoralism resources and the reestablishment of wetter conditions could have triggered the simultaneous population expansions observed here. R-V88 also shows signals of a further and more recent (~ 5.5 kya) Saharan demographic expansion which involved the R-V1589 internal clade. We observed similar demographic patterns in all the other haplogroups in about the same period and in different geographic areas (A3-M13/V3, E-M2/V3862 and E-M78/V32 in the Horn of Africa, E-M2/M191 in the central Sahel/central Africa), in line with the hypothesis that the start of the desertification may have caused massive economic, demographic and social changes [1].

Finally, the onset of the arid conditions at the end of the last African humid period was more abrupt in the eastern Sahara compared to the central Sahara, where an extensive hydrogeological network buffered the climatic changes, which were not complete before ~ 4 kya [6, 62, 64]. Consistent with these local climatic differences, we observed slight differences among the four trans-Saharan haplogroups. Indeed, we found that the contact between northern and sub-Saharan Africa went on until ~ 4.5 kya in the central Sahara, where we mainly found the internal lineages of E-M2 and R-V88 (Additional file 2: Figures S4 and S6). In the eastern Sahara, we found a sharper and more ancient (> 5 kya) differentiation between the people from northern Africa (and, more generally, from the Mediterranean area) and the groups from the eastern sub-Saharan regions (mainly from the Horn of Africa), as testified by the distribution and the coalescence ages of the A3-M13 and E-M78 lineages (Additional file 2: Figures S3 and S5).

## Conclusions

Our data suggest that the favourable climatic conditions and the fertile environment established in the Sahara during the last African humid period promoted the occupation and dispersal of human groups and contributed to the present distribution of Y lineages in northern and sub-Saharan Africa. On the contrary, historical events, such as the Arab slave trade, had only a marginal role in the Y genetic composition of African people. Our inferences are based on the assumption that migration events that occurred in recent times are reflected by recent coalescence times of Y lineages. To support our assumption, we included in this study a large number of African, European and near-eastern ethnic groups (both from our lab collection and from the literature data) in order to consider as much genetic diversity as possible. However, we could not completely exclude the presence of informative haplogroups in other populations/regions absent in our sample collection. More sample efforts could lead to the identification of other informative data from other populations and these findings could refine our inferences. Nonetheless, this study highlights the importance of the targeted selection and analysis of uniparental haplogroups with a relic distribution to understand past human history, which could be concealed by the genetic changes caused by successive events.

## Methods

### The sample

We performed targeted NGS on 104 subjects from our lab collection selected on the basis of their haplogroup affiliation [19, 22, 33–35, 37, 52, 65], focusing on the four trans-Saharan haplogroups A3-M13, E-M2, E-M78 and R-V88. In order to increase the power of resolution of the study, from the literature we also added 42 Y chromosomes sequenced at high-coverage [45, 48]. Finally, we included four radiocarbon-dated ancient specimens [46, 47, 49] to be used as calibration points for the time estimates, for a total of 150 subjects (Additional file 1: Table S1). We also selected 142 informative markers to genotype more than 6000 men belonging to 128 populations (see below).

### DNA quality control

The 104 DNA samples from our lab collection were obtained from peripheral blood, saliva or cultured cells. Target sequencing required specific quality and quantity parameters for the DNA to be analysed: 1) absence or low amount of DNA degradation; 2) quantity ≥ 3 μg; 3) concentration ≥ 37.5 ng/μl; 4) purity, A260/280 = 1.8–2.0. Concentration and purity were measured using a NanoDrop 1000 spectrophotometer, produced by Thermo Fisher Scientific. Degradation was assessed by means of an electrophoretic run on a 1 % agarose gel. We performed a whole genome amplification (WGA) of 59 samples with an insufficient quantity of DNA, using the GenomiPhi V2 DNA Amplification kit (GE Healthcare) according to the manufacturer’s protocol.

### Selection of the unique MSY regions to be sequenced

We selected 22 blocks within the X-degenerate portion of the Y chromosome [66] (Fig. 1; Additional file 1: Table S6), for a total of about 11 Mb which were characterized by a low degree of homology with the X chromosome or with the autosomes [67, 68]. The total number of targeted bases decreased to about 4 Mb after the exclusion of the repetitive elements [69]. For these selection steps, we used the “Table browser” tool of the UCSC Genome browser, considering the aligned annotation tracks for the human February 2009 (GRCh37/hg19) assembly.

### Targeted NGS

Library preparation, targeting, sequencing and alignment were performed by BGI-Tech (Hong Kong). The targeted unique regions of the MSY were captured using a Roche Nimblegen custom capture array, composed of a set of 200-bp probes. The probes excluded almost all the repetitive elements from the 22 X-degenerated blocks, capturing a total of about 4.4 Mb. The captured regions were loaded onto an Illumina HiSeq 2500 platform to produce a > 50× mean depth for the targeted 4.4 Mb.

The low quality reads, contamination with adapters and repeated reads were discarded and the sequences of each subject aligned to the human Y chromosome reference sequence (GRCh37/hg19) by means of the BWA (Burrows-Wheeler Aligner) software [70], generating an alignment file (.bam format) [71, 72].

### Selection of the final set of reliable bases

In order to discard problematic regions (involved in rearrangements, deletions, duplication, etc.) and to obtain a reliable set of bases for the SNP calling in all 104 subjects, we performed an analysis of depth through the extraction of some informative values from each .bam file using the SAMtools platform [71, 73] (Additional file 3: Supplementary Text). In this way, we obtained a final set of ~ 3.3 Mb, which were used for all the subsequent analysis (Additional file 1: Table S7).

### SNP calling and filtering

The variant positions were extracted using the SAMtools platform [71, 73] by comparing our 104 sequences to the human Y chromosome reference sequence (February 2009, GRCh37/hg19 assembly). The output was in the form of a VCF (Variant Call Format) file for each sample. The same process was performed for the Y chromosome of the ancient samples [46, 47, 49]. On the contrary, for the 42 modern public subjects from Complete Genomics [45] and Karmin and colleagues [48], we extracted the variant positions within the final ~ 3.3 Mb directly from publicly available VCF files.

In order to discard false positive calls, we applied different filtering criteria, which can be grouped into three different categories: 1) direct filtering—we used the information embedded in the VCF file to accept or discard the variant positions; 2) manual filtering—we manually checked the uncertain cases from the previous filtering step in the alignment (.bam) files [72]; 3) cluster filtering—we checked for clusters of SNPs (i.e. groups of two or more SNPs occurring in close proximity and on the same branch of the Y phylogeny) and decided whether to maintain or discard them from the analyses (Additional file 3: Supplementary Text).

### Tree reconstruction and validation

The maximum parsimony phylogenetic tree was reconstructed using the MEGA software [74]. Because we did not assign univocally to A00 or A0-T the mutational events on branch 1, the tree root was positioned by default to the midpoint (Additional file 3: Supplementary Text). We identified 25 recurring mutations, 11 triallelic variants and two variant positions whose direction cannot be assigned on the basis of the phylogeny (Additional file 1: Tables S2 and S8 and Additional file 3: Supplementary Text). All these positions were accurately checked in the alignment files. The presence in our list of already identified variants in published papers [35, 48, 50–53, 57] and in the ISOGG dataset [75] made it possible for us to check the efficiency of all the steps from the SNP calling to the tree reconstruction. Our data successfully passed all these control levels.

### Mutation rate, dating and star-like index

The estimate of the mutation rate was obtained using the BEAST software [76]. The input file (nexus format) was loaded onto the BEAUTY suite and we assigned to the four ancient samples the calibrated radiocarbon dates, expressed in years before present (BP): 1) Loschbour [46], 8055 years BP; 2) Kotias [49], 9712 years BP; 3) Bichon [49], 13,665 years BP; 4) Ust’-Ishim [47], 44,890 years BP. We used a GTR nucleotide substitution model under a strict clock or a lognormal relaxed clock and an expansion growth model for the population size, using parameters set as in Trombetta et al. [52]. The output was checked with the Tree Annotator and Tracer platforms. The mutation rate for the ~ 3.3 Mb analysed here was 0.735 ± 0.03 × 10^−9^/site/year, corresponding to about one new mutational event every 408 years.

We applied different methods to estimate the age of the nodes of the tree on the basis of the available information for each node. The nodes of the tree obtained from the NGS data have been dated using both the method implemented by BEAST (with the parameters described above under a strict clock) and the Rho statistics, since we knew the precise number of SNPs downstream of each node. The Rho statistic, its associated standard deviation and the corresponding values expressed in years have been calculated using the Network software [77] (Table 1).

Since we lacked complete information regarding the number of SNPs downstream of the nodes identified from the genotyping, it was not possible to use the Rho statistic to date the new internal nodes. In these cases, we applied two different methods, using the genotyping information of the SNPs on the split branches or extrapolating it from the 1000 Genomes Project [51] Y sequences (Additional file 1: Table S9 and Additional file 3: Supplementary Text).

We used the Rho statistic and its standard deviation to calculate the star-like index of the tree nodes, according to the formula ρ/(*n* × SD^2^), where ρ is the Rho value for the considered node, SD is the Rho standard deviation and *n* is the number of tips downstream of the node [78, 79]. The star-like index can take values between 1/*n* and 1, where 1 corresponds to a perfect star-like topology, characterised by sister lineages splitting from the same node. Usually, values ≥ 0.5 are considered high star-like indexes [80].

### Population analysis

We selected a total of 142 informative polymorphisms to be genotyped in the whole set of 6065 men from the 128 populations of our lab collection (Fig. 4; Additional file 1: Table S5). The 142 SNPs (including 21 known variants which did not fall within our NGS target region) were chosen on the basis of their phylogenetic position and ethno-geographic distribution, also considering other datasets [35, 48, 50–53, 57] and in the ISOGG [75] (Additional file 1: Table S4). The chosen SNPs have been analysed by PCR and Sanger sequencing or RFLP. Moreover, we extracted the frequency distribution of the selected variants from the NGS data of one Sardinian population [53] and 16 populations from phase 3 of the 1000 Genomes Project [51] (Fig. 4; Additional file 1: Table S5).

### Frequency maps

Frequency maps were drawn on a grid with 100 rows × 78 columns using the Kriging method implemented by the Surfer 6.0 software (Golden Software, Inc., Golden, CO, USA). We used the frequency data of all the African and western Eurasian populations (Fig. 4).

## Additional files

Additional file 1: **Table S1.** List of samples analysed by NGS. **Table S2.** List of variant positions in the whole set of 150 Y chromosomes. **Table S3.** Time estimates (kya) for the nodes of Fig. 2a, obtained with BEAST assuming both a strict and a relaxed clock. **Table S4.** List of SNPs used for the molecular dissection and population analysis of the four trans-Saharan clades. **Table S5.** Relative frequencies (percentage) of the A3-M13, E-M2, E-M78 and R-V88 sub-haplogroups in the 145 populations analyzed. **Table S6.** Twenty-two blocks of the X-degenerate portion of the MSY targeted for the NGS. **Table S7.** List of the unique fragments of the capture probe set covering the 22 selected MSY regions. **Table S8.** Description of the two unassigned variant positions. **Table S9.** Branch assignment of the SNPs that have been extracted from the literature. (XLSX 1131 kb)

Additional file 2: **Figure S1.** Eulero-Venn diagram representing the proportion of shared variants between the present study and four recently published papers. **Figure S2.** Comparison between Rho and BEAST dating methods. **Figure S3.** A3-M13 phylogeny and distribution. **Figure S4.** E-M2 phylogeny and distribution. **Figure S5.** E-M78 phylogeny and distribution. **Figure S6.** R-V88 phylogeny and distribution. (PDF 472 kb)

Additional file 3: Supplementary Text. (DOCX 73 kb)

## Abbreviations

BP: Before present
kya: Thousand years ago
Mb: Megabase
MSY: Male-specific portion of the human Y chromosome
NGS: Next generation sequencing
RFLP: Restriction fragment length polymorphism
SNP: Single nucleotide polymorphism
VCF: Variant call format
WGA: Whole genome amplification
