# Supplementary material for: The peopling of the last Green Sahara revealed by high-coverage resequencing of trans-Saharan patrilineages
Source: Genome Biol. 2018 Feb 12;19:20. doi: 10.1186/s13059-018-1393-5 (PMC5809971; doi:10.1186/s13059-018-1393-5)
Supplement: Supplementary file 2 — Figure S1. Eulero-Venn diagram representing the proportion of shared variants between the present study and four recently published papers. Figure S2. Comparison between Rho and BEAST dating methods. Figure S3. A3-M13 phylogeny and distribution. Figure S4. E-M2 phylogeny and distribution. Figure S5. E-M78 phylogeny and distribution. Figure S6. R-V88 phylogeny and distribution. (PDF 472 kb) [file 13059_2018_1393_MOESM2_ESM.pdf]

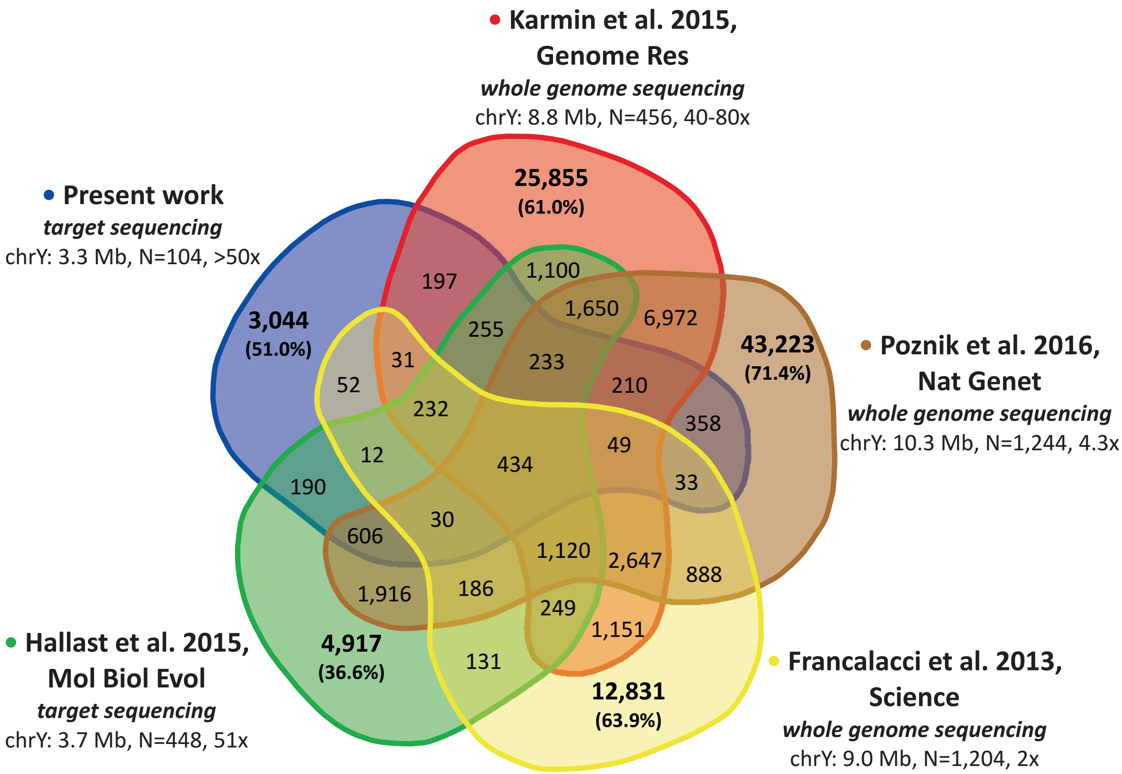

Supplementary Figure S1. Eulero-Venn diagram representing the proportion of shared variants between present study and four recently published papers. For each of the four published studies , we reported: the sequencing approach, the amount of sequenced bases on the Y chromosome, the number of analysed subjects and the average depth of the sequences. Please note: the areas of the diagram are not proportional to the number of variants.

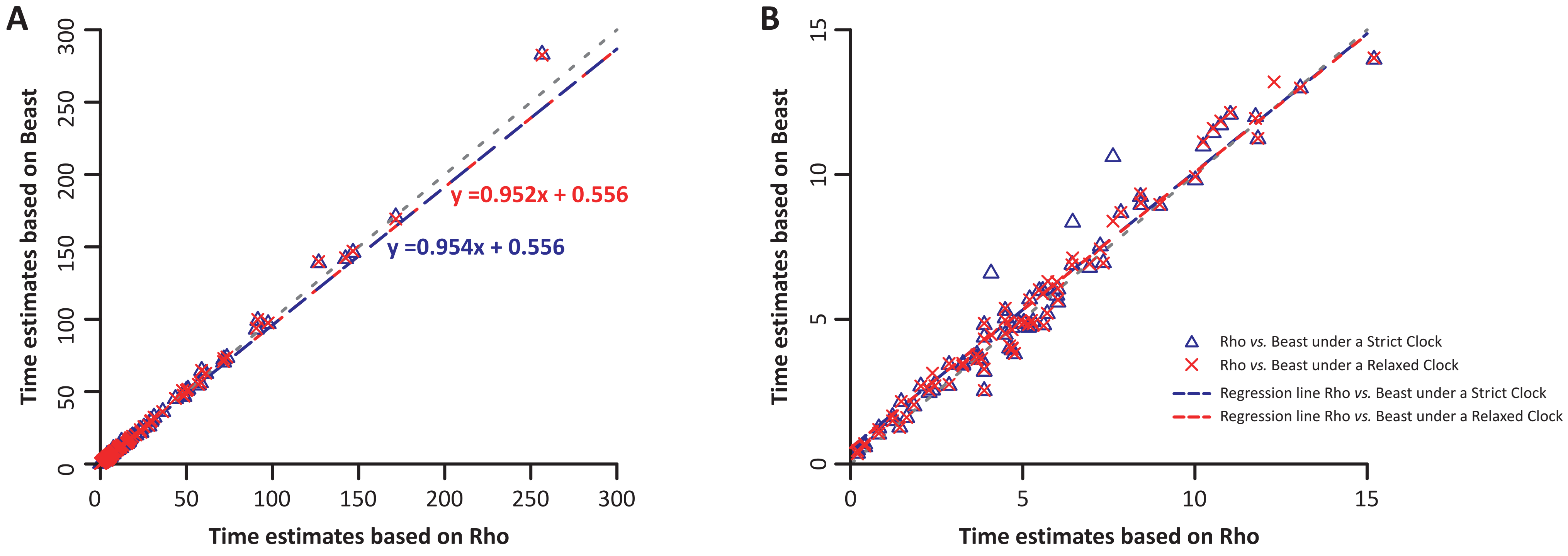

Supplementary Figure S2. Comparison between Rho and BEAST dating methods. (**A**) Comparison between the time estimates based on the Rho method and the time estimates obtained with BEAST, assuming both a strict clock (blue) and a relaxed clock (red). **(B)** A detail of panel A, including only the time estimates ≤15 kya.

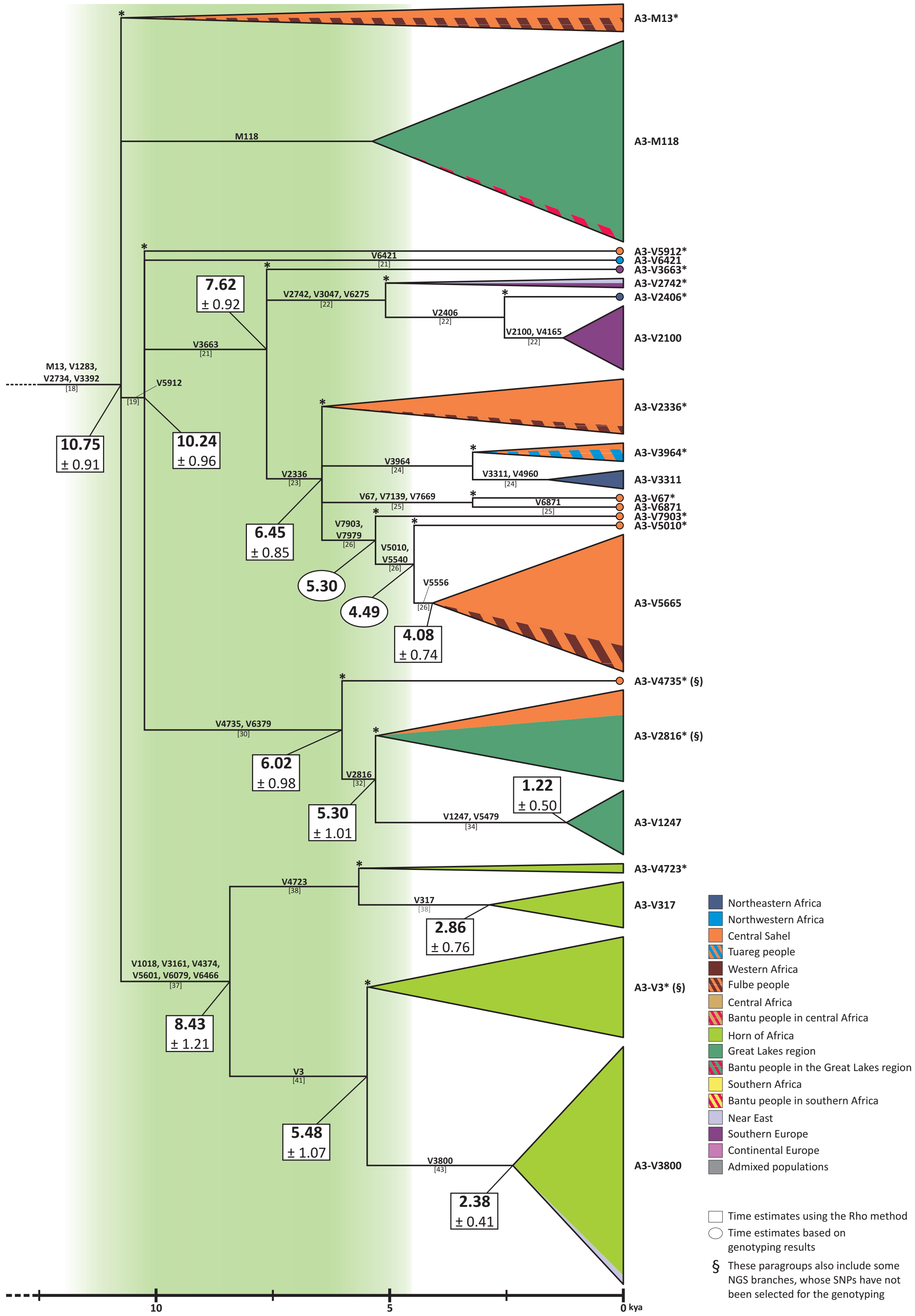

Supplementary Figure S3. A3-M13 phylogeny and distribution. Branch lengths and triangle widths are drawn proportional to the estimated times between successive splits (timeline at the bottom). For the nodes whose age could not be estimated, we drew the split at the midpoint of the branch. The height of each triangle is proportional to the corresponding sample size and coloured according to the samples' place of origin. Single-sample lineages are represented by a coloured circle. The last Green Sahara period is highlighted by a green belt in the background.

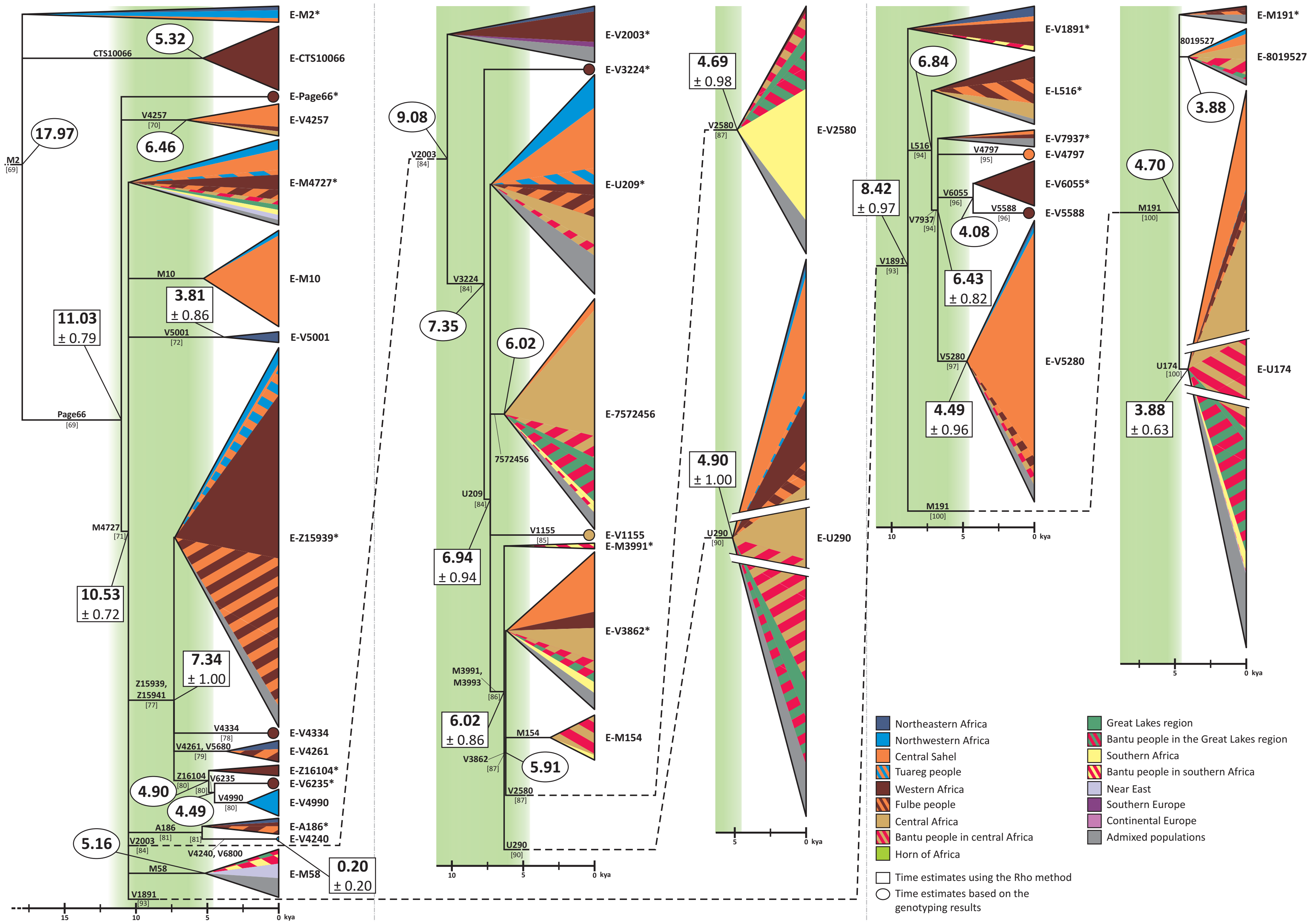

Supplementary Figure S4: E-M2 phylogeny and distribution. Branch lengths and triangle widths are drawn proportional to the estimated times between successive splits (timeline at the bottom). For the nodes whose age could not be estimated, we drew the split at the midpoint of the branch. The height of each triangle is proportional to the corresponding sample size and coloured according to the samples' place of origin. Single-sample lineages are represented by a coloured circle. The last Green Sahara period is highlighted by a green belt in the background.

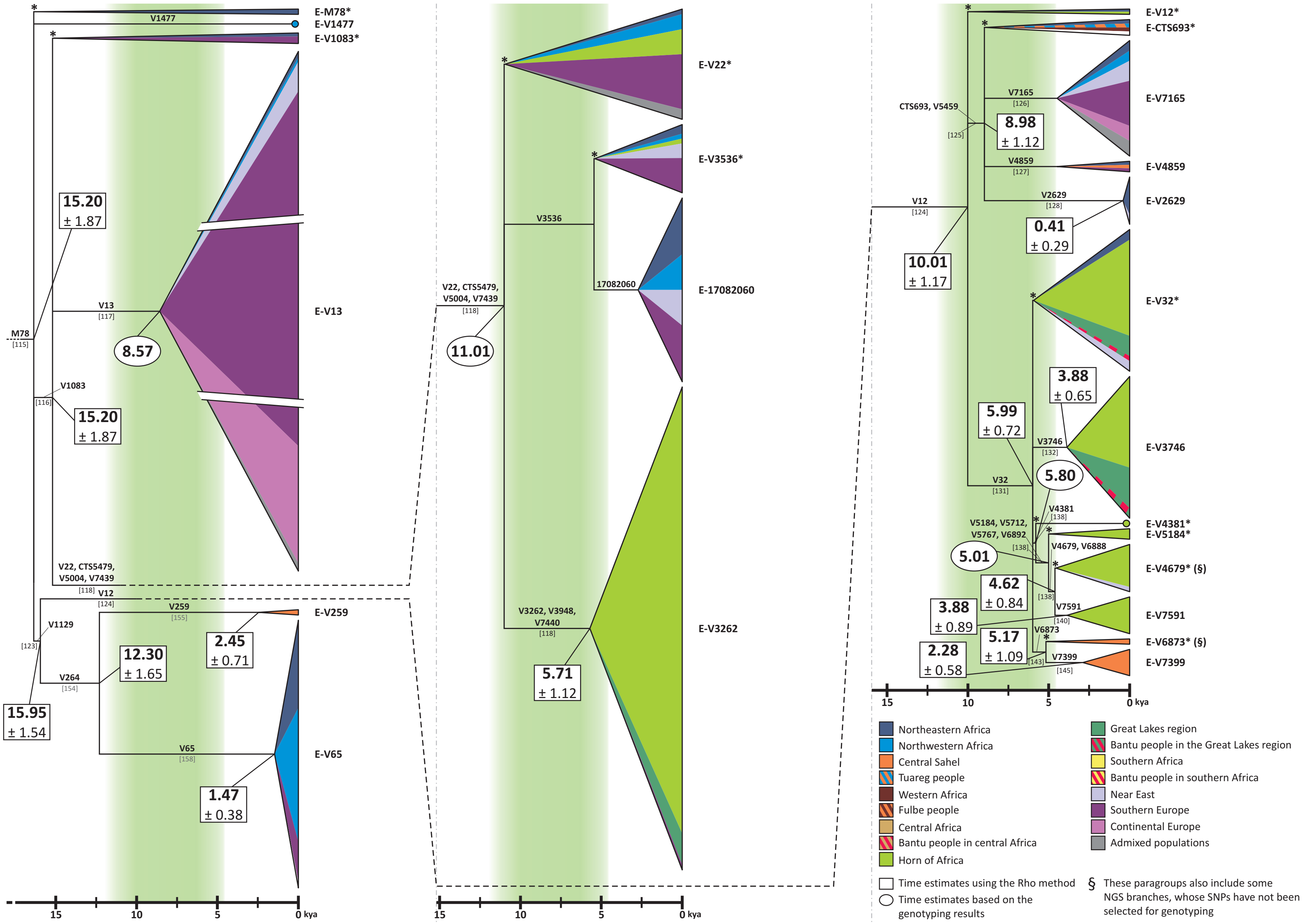

Supplementary Figure S5: E-M78 phylogeny and distribution. Branch lengths and triangle widths are drawn proportional to the estimated times between successive splits (timeline at the bottom). For the nodes whose age could not be estimated, we drew the split at the midpoint of the branch. The height of each triangle is proportional to the corresponding sample size and coloured according to the samples' place of origin. Single-sample lineages are represented by a coloured circle. The last Green Sahara period is highlighted by a green belt in the background.

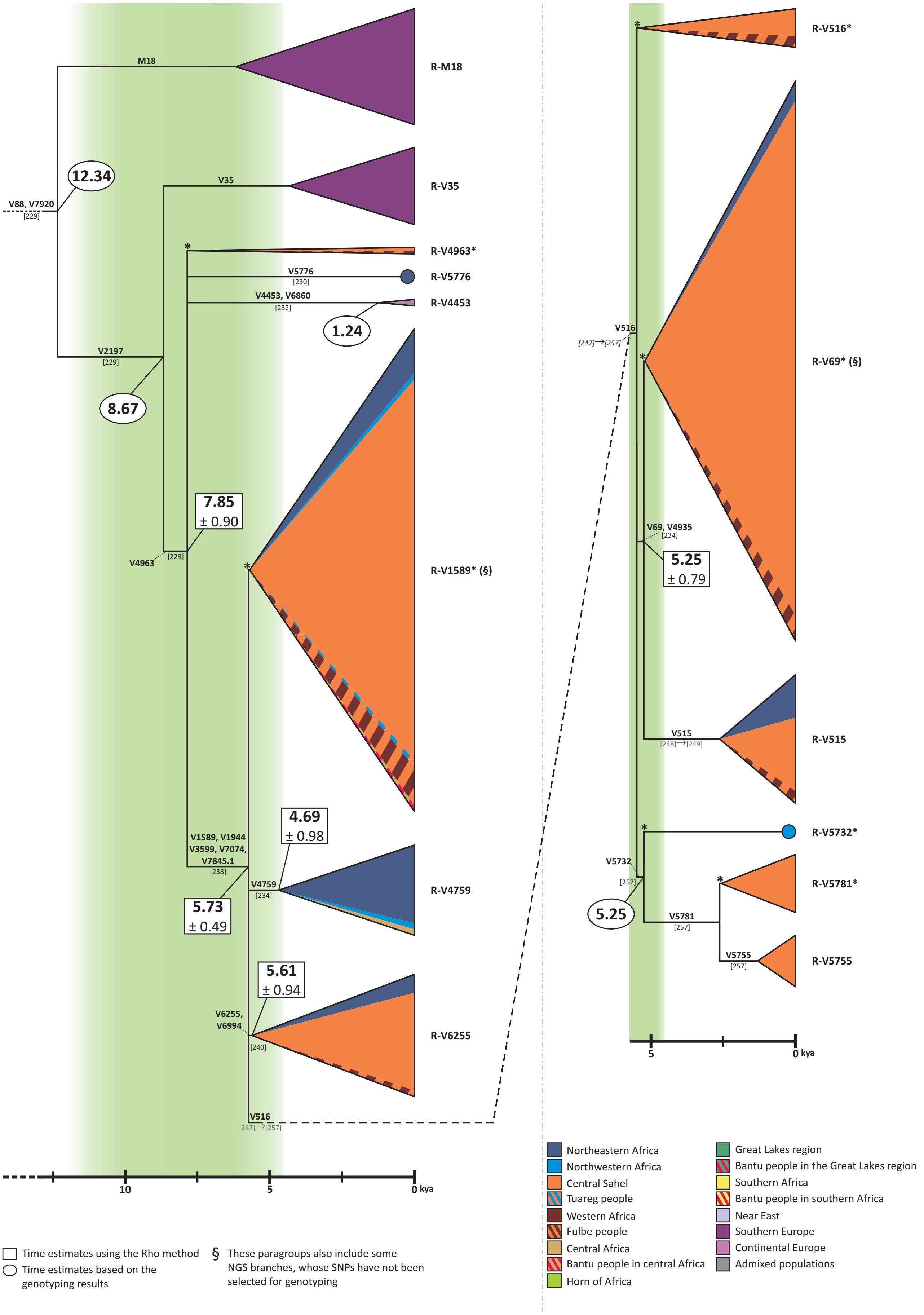

Supplementary Figure S6: R-V88 phylogeny and distribution. Branch lengths and triangle widths are drawn proportional to the estimated times between successive splits (timeline at the bottom). For the nodes whose age could not be estimated, we drew the split at the midpoint of the branch. The height of each triangle is proportional to the corresponding sample size and coloured according to the samples' place of origin. Single-sample lineages are represented by a coloured circle. The last Green Sahara period is highlighted by a green belt in the background.
