# Supplementary material for: The peopling of the last Green Sahara revealed by high-coverage resequencing of trans-Saharan patrilineages
Source: Genome Biol. 2018 Feb 12;19:20. doi: 10.1186/s13059-018-1393-5 (PMC5809971; doi:10.1186/s13059-018-1393-5)
Supplement: Supplementary file 3 — Supplementary Text. (DOCX 73 kb) [file 13059_2018_1393_MOESM3_ESM.docx]

Supplementary Text

[1. Filtering of the deep-sequenced bases 2](#_Toc500600526)

[*1.1 Analysis of the depth* 2](#_Toc500600527)

[*1.2 Analysis of putative deletions/duplications* 2](#_Toc500600528)

[2. SNP filtering 3](#_Toc500600529)

[*2.1 Direct filtering* 3](#_Toc500600530)

[*2.2 Manual filtering* 5](#_Toc500600531)

[*2.3 Cluster filtering* 5](#_Toc500600532)

[3. Reconstruction of the Y chromosome phylogenetic tree 5](#_Toc500600533)

[4. Time estimates of the nodes obtained from the genotyping analysis 6](#_Toc500600534)

[Supplementary Tables 8](#_Toc500600535)

[Supplementary Table 1. 8](#_Toc500600536)

[Supplementary Table 2. 8](#_Toc500600537)

[Supplementary Table 3. 8](#_Toc500600538)

[Supplementary Table 4. 8](#_Toc500600539)

[Supplementary Table 5. 8](#_Toc500600540)

[Supplementary Table 6. 9](#_Toc500600541)

[Supplementary Table 7. 9](#_Toc500600542)

[Supplementary Table 8. 9](#_Toc500600543)

[Supplementary Table 9. 9](#_Toc500600544)

[Supplementary Figures 10](#_Toc500600545)

[Supplementary Figure 1. 10](#_Toc500600546)

[Supplementary Figure 2. 10](#_Toc500600547)

[Supplementary Figure 3. 10](#_Toc500600548)

[Supplementary Figure 4. 10](#_Toc500600549)

[Supplementary Figure 5. 11](#_Toc500600550)

[Supplementary Figure 6. 11](#_Toc500600551)

[References 12](#_Toc500600552)

# 1. Filtering of the deep-sequenced bases

## *1.1 Analysis of the depth*

Due to the possible imbalance in the amplification of the regions introduced by the WGA (Whole Genome Amplification) method, we analysed the 59 WGA samples separately from the 45 genomic DNA samples. For both datasets, using SAMtools [1,2], we extracted the depth value for each base of the 22 MSY captured regions (Fig. 1 and Supplementary Table S6), after excluding the reads with low mapping quality (MQ < 30). Successively, we calculated the moving average of the mean depth per position along the entire captured region, using sliding windows of 1000 bp moving 1 bp. Then, we discarded all the positions with a mean depth value which was in the lower 3‰ range of the average depth distribution in the genomic dataset or in the lower 4‰ range in the WGA dataset. We also discarded two blocks (chrY: 21152803-21154906; chrY: 28793241-28819317) with very high average depth values in the genomic dataset, because these values could be indicative of Y chromosome rearrangements. After the depth analysis, we excluded 29,135 bp. We further refined the filtered regions removing ~ 0.70 Mb within the repetitive elements (using the Repeat Masker and Simple Repeats tracks from the Table browser tool of the UCSC Genome Browser).

We obtained a total of ~ 3.7 Mb which passed our quality and depth controls.

## *1.2 Analysis of putative deletions/duplications*

We also used the depth information to identify candidate deletions or duplications within our target regions on the Y chromosome. We extracted the raw depth (without filtering for the MQ) for each of the 3.7 Mb obtained from the previous filtering steps. For each subject, we performed the moving average using sliding windows of 100 bp moving 1 bp. Then, we separately analysed genomic and WGA samples, choosing for each dataset an arbitrary reference sample with a good coverage (S178 and S179, for the genomic and WGA group, respectively). For both datasets, the average depth value of each 100 bp interval was divided by the corresponding depth value of the reference sample. The identification of continuous clusters of positions with a ratio value ≤ 0.05 in at least one subject were marked as putative deletions. On the contrary, putative deletions on the reference sample led to a very high (theoretically infinite) ratio in all the other samples.

The identification of putative duplications with the same method was difficult because of the strong oscillations of the observed depth value. Thus, we applied a double standardization method, dividing the moving average depth values by the total average depth of the same sample and then by the corresponding value of the reference subject. Despite this, the fluctuations were too strong to obtain reliable data in the WGA dataset, so we could only analyse the data from the group of the genomic samples. We identified clusters of positions with a value ratio ≥ 1.8 that has been considered to be indicative of a duplication. Putative duplications on the reference sample, on the other hand, led to a very low (theoretically zero) ratio in all the samples. All these situations were checked on the .bam files.

Through this approach, we were able to identify ~ 0.36 Mb possibly involved in deletions or duplications, which need to be experimentally validated. However, these regions show sub-optimal depth parameters, so we decided to discard them.

After all these analyses, we obtained a final set of 3,328,701 Mb of unique and reliable Y chromosome regions (Supplementary Table S7).

These ~ 3.3 Mb were also extracted from the Y chromosome alignment files (.bam) of the four ancient subjects [3–5].

# 2. SNP filtering

## *2.1 Direct filtering*

We analysed the variant positions identified in the three different datasets separately: 1) our 104 sequenced subjects; 2) the 42 publicly available complete Y chromosomes [6,7] and the 4 ancient specimens [3–5].

The 104 samples sequenced in the present study

The parameters considered were the quality (“QUAL” field), the depth (“DP” field) and the number of reads with the reference or the alternative base (“DP4” values), all extracted from the VCF file of each sample. Using the information in the DP4, we calculated a new parameter used for a more accurate filtering:

$$FilDP4= \frac{Number of reads with the ALT base -Number of reads with the REF base}{Total number of reads}$$

We directly discarded variant positions with FilDP4 ≤ 0.3 and retained all the SNPs with FilDP4 > 0.8 and QUAL ≥ 100. In the other cases, we considered the known phylogenetic context. For SNPs shared among samples belonging to the same monophyletic cluster, we applied less severe criteria (FilDP4 ≥ 0.6 and the number of ALT reads ≥ 2), while we discarded the private positions with DP < 2 or FilDP4 and DP less than 0.4 and 4, respectively. The remaining cases have been manually checked in the alignment file.

The 42 public Y high-coverage sequences

For these samples [6,7], we could not check the uncertain positions on the .bam files, so we applied more restrictive criteria for the filtering to avoid false positives. Their VCF files have a different structure, so we used different parameters for the filtering, namely FT and AD. The former indicates if the position passed (“PASS”) or not (“VQLOW”) all the filtering criteria used for the SNP calling, while the latter reports the number of reads with the alternative base. If FT = VQLOW or AD ≤ 2, the presence of the same variant position in other phylogenetically related samples was checked: if present, the SNP was retained, otherwise it was discarded. For all the other possible values of FT and AD, the SNPs have been considered reliable.

The 4 ancient specimens

The four ancient Y chromosomes come from different studies, so they show different features. For this reason, they have been analysed separately. The Ust’-Ishim [3] and the Loschbour [4] samples have a quite high quality/depth, so the same filtering criteria applied for our 104 samples have been used (see above).

On the contrary, Kotias and Bichon [5] have been found to be more critical, so we defined specific filtering criteria. If FilDP4 ≤ 0 or DP < 2, the position was discarded, while it was accepted if ALT ≥ 2 and FilDP4 > 0.4. For the intermediate cases, the presence of the same variant position in other subjects was checked. Private positions were discarded if ALT < 2 or FILDP4 and DP less than 0.4 and 4, respectively. The other cases have been manually checked in the alignment files.

## *2.2 Manual filtering*

All the unresolved cases from the direct filtering have been manually checked in the alignment .bam file of the samples of interest. In the final decision, we considered several criteria such as phylogenetic context, the depth and the quality of the region in all the subjects, the proximity of repetitive elements or short indels, the presence of the same variant position with suboptimal parameters in other subjects and the mapping quality of the reads.

## *2.3 Cluster filtering*

We checked for the presence of SNP clusters (with each cluster made up of 2 or more mutations), occurring in the same phylogenetic context and at closely spaced positions (less than 20 bp), in order to discard the groups of SNPs that could have been generated by the same mutational event (for example, as a consequence of gene conversion). When the cluster was composed of 3 or more SNPs or of 2 polymorphisms separated by only one or two bp, the involved variant positions were discarded.

At the end of the filtering process, 5,966 SNPs passed all the criteria in our 104 subjects. The number increased to 7,544 SNPs in the whole set of 150 samples (Supplementary Table S2).

# 3. Reconstruction of the Y chromosome phylogenetic tree

Considering the phylogenetic relations among the 150 subjects, we were able to recognise the ancestral and the derived state for most of the 7,544 variant positions and, consequently, it has been possible to assign them univocally to one branch (or more branches, in cases of recurrent variants) (Supplementary Table S2).

However, in some cases, we could not infer the direction of the mutational event. 857 positions (including one triallelic and three recurrent mutations) are different from the reference in all the samples except for the A00 subject. The difference from the reference of the A00 lineage can put down to either an A00-specific mutational event or a mutation which occurred at the root of A0-T. For this reason, we defined these two branches together as “branch 1” and the resulting tree is unrooted (Fig. 2, panel a).

Moreover, we identified two recurrent mutations (V1233.1 and V5349) which could be interpreted in two different ways (Supplementary Table S2 and Fig. 2, panel a). V1233.1 has been assigned to branches 1 and 6 looking at the corresponding position on the chimpanzee Y chromosome, assuming that the chimpanzee maintained the ancestral state. V5349 could not be assigned considering the chimpanzee sequence (in red in Supplementary Table S8). We decided to interpret it as a mutation in branch 12 and a reversion in branch 18, considering two different possible explanations for this pattern:

1) the mutation “T” to “C” on branch 12 would form a CpG site; CpG sites show a higher mutation rate compared to the rest of the genome [8,9], so our position could have experienced a reversion in branch 18;

2) the variant position falls within a Y portion showing about 90% of similarity with the X chromosome, which has a “T” in the corresponding position; as a consequence, the reversion on branch 18 could also be explained as the result of a X-to-Y gene conversion event.

We also identified 11 triallelic variants and 23 recurring mutations. Among these, one is found three times in our phylogeny, four can be recognised as reversions and two can be interpreted either as double-hit mutations or reversions because one of the branches involved is branch 1 and so these SNPs cannot be univocally assigned. Finally, we identified 21 polymorphisms which were different from the reference sequence but invariant in all the samples. These variants have been interpreted as reference-specific mutations (Supplementary Table S2).

# 4. Time estimates of the nodes obtained from the genotyping analysis

For the lineages identified after the genotyping step (Supplementary Figs. S3-S6), we did not have the information about their variant positions, so we applied two different approaches to estimate their coalescence age. Let *n* be the new node identified:

1) When we exactly knew the number of markers in the branch upstream *n*, the number of mutations in the NGS branch downstream *n* and if the node downstream (*r*) was precisely dated by means of the rho statistics, we simply counted the number of SNPs from *n* to *r* and multiplied the total by 408 years. We then added this time to the age of *r* to obtain the time estimate of *n*. The complete information about the number of SNPs downstream and upstream *n* was obtained by the genotyping of all the markers defining the involved branch (Supplementary Table S3) or from literature [10,11] (Supplementary Table S9).

2) If *n* was also found in the phylogeny produced by the Phase 3 of the 1000 Genomes Project [12] and there were no precisely dated downstream nodes to be used as a reference, we estimated the age of *n* by measuring its height, with the traversing approach used by Poznik and collegues [11]. We measured the distances (*d*), expressed in number of SNPs, among three nodes: the node to be dated (*n*), a reference node precisely dated with the rho method (*r*) and an ancestor node between them (*a*).

$$d_{rn}= d_{ra}- d_{an}$$

We then multiplied this value by 408 years and this time was summed to the age of the reference node to obtain the age of *n*.

# Supplementary Tables

*(provided in a separate Excel file: Additional file 1)*

Supplementary Table 1. List of samples analysed by NGS. All the samples used for the phylogenetic tree reconstruction (both from this study and from the literature) are reported, along with information about their haplogroup affiliation, their age (modern or ancient) and their ethno-geographic origin.

Supplementary Table 2. List of variant positions in the whole set of 150 Y chromosomes. For each biallelic polymorphism, we reported: the coordinate on the Y chromosome, the reference and alternative base, the position in the tree (branch number and haplogroup), the information from other studies/public databases and specific notes for special cases.

### Supplementary Table 3. Supplementary Table 3. Time estimates (kya) for the nodes of fig. 2, panel A, obtained with BEAST assuming both a strict and a relaxed clock. For each node, we reported the time estimates and their 95% highest posterior density (HPD) interval, obtained with BEAST assuming two different clock parameters.

Supplementary Table 4. List of SNPs used for the molecular dissection and population analysis of the four trans-Saharan clades. The list is ordered by haplogroup/branch and all the informative data about each SNP are reported.

Supplementary Table 5. Relative frequencies (%) of the A3-M13, E-M2, E-M78 and R-V88 sub-haplogroups in the 145 populations analysed. For each population we reported: ID number, name of the population, reference, total number of analysed Y chromosomes (N), country of origin, macro-region classification (used in Fig. 2 and Supplementary Figs. 3-6), linguistic affiliation and sub-haplogroup relative frequencies.

Supplementary Table 6. Twenty-two blocks of the X-degenerate portion of the MSY targeted for the NGS. The coordinates and the size of each block are reported.

Supplementary Table 7. List of the unique fragments of the capture probe set covering the 22 selected MSY regions. The coordinates and the size of each fragment are reported.

Supplementary Table 8. Description of the two unassigned variant positions. The variant position which could not be assigned considering the chimpanzee sequence is labelled in red.

Supplementary Table 9. Branch assignment of the SNPs that have been extracted from the literature. These variants were not manually genotyped because we could extract their haplogroup information from the literature. The Y coordinate, the direction, the position on the tree before and after the genotyping, the corresponding Supplementary figure and the reference are reported for each SNP.

# Supplementary Figures

*(provided in a separate PDF file: Additional file 2)*

## Supplementary Figure 1. Eulero-Venn diagram representing the proportion of shared variants between present study and four recently published papers. For each of the four published studies [7,11,13,14], we reported: the sequencing approach, the amount of sequenced bases on the Y chromosome, the number of analysed subjects and the average depth of the sequences. Please note: the areas of the diagram are not proportional to the number of variants.

## Supplementary Figure 2. Comparison between rho and BEAST dating methods. On the axis, the time estimates for the nodes of fig. 2 obtained with the two different approaches.

## Supplementary Figure 3. A3-M13 phylogeny and distribution. Branch lengths and triangle widths are drawn proportional to the estimated times between successive splits (timeline at the bottom). For the nodes whose age could not be estimated, we drew the split at the midpoint of the branch. The height of each triangle is proportional to the corresponding sample size and coloured according to the samples’ place of origin. Single-sample lineages are represented by a coloured circle. The last Green Sahara period is highlighted by a green belt in the background.

## **Supplementary Figure 4. E-M2 phylogeny and distribution.** Branch lengths and triangle widths are drawn proportional to the estimated times between successive splits (timeline at the bottom). For the nodes whose age could not be estimated, we drew the split at the midpoint of the branch. The height of each triangle is proportional to the corresponding sample size and coloured according to the samples’ place of origin. Single-sample lineages are represented by a coloured circle. The last Green Sahara period is highlighted by a green belt in the background.

## **Supplementary Figure 5. E-M78 phylogeny and distribution.** Branch lengths and triangle widths are drawn proportional to the estimated times between successive splits (timeline at the bottom). For the nodes whose age could not be estimated, we drew the split at the midpoint of the branch. The height of each triangle is proportional to the corresponding sample size and coloured according to the samples’ place of origin. Single-sample lineages are represented by a coloured circle. The last Green Sahara period is highlighted by a green belt in the background.

## **Supplementary Figure 6. R-V88 phylogeny and distribution.** Branch lengths and triangle widths are drawn proportional to the estimated times between successive splits (timeline at the bottom). For the nodes whose age could not be estimated, we drew the split at the midpoint of the branch. The height of each triangle is proportional to the corresponding sample size and coloured according to the samples’ place of origin. Single-sample lineages are represented by a coloured circle. The last Green Sahara period is highlighted by a green belt in the background.

# References

1. Li H, Handsaker B, Wysoker A, Fennell T, Ruan J, Homer N, et al. The Sequence Alignment/Map format and SAMtools. Bioinformatics. 2009;25:2078–9.

2. Li H. A statistical framework for SNP calling, mutation discovery, association mapping and population genetical parameter estimation from sequencing data. Bioinformatics. 2011;27:2987–93.

3. Fu Q, Li H, Moorjani P, Jay F, Slepchenko SM, Bondarev AA, et al. Genome sequence of a 45,000-year-old modern human from western Siberia. Nature. Nature Publishing Group; 2014;514:445–9.

4. Lazaridis I, Patterson N, Mittnik A, Renaud G, Mallick S, Kirsanow K, et al. Ancient human genomes suggest three ancestral populations for present-day Europeans. Nature. Nature Publishing Group; 2014;513:409–13.

5. Jones ER, Gonzalez-Fortes G, Connell S, Siska V, Eriksson A, Martiniano R, et al. Upper Palaeolithic genomes reveal deep roots of modern Eurasians. Nat. Commun. 2015;6:8912.

6. Drmanac R, Sparks A, Callow M. Human genome sequencing using unchained base reads on self-assembling DNA nanoarrays. Science. 2010;327:78–81.

7. Karmin M, Saag L, Vicente M, Sayres MAW, Järve M, Talas UG, et al. A recent bottleneck of Y chromosome diversity coincides with a global change in culture. Genome Res. Cold Spring Harbor Laboratory Press; 2015;25:459–66.

8. Ségurel L, Wyman MJ, Przeworski M. Determinants of Mutation Rate Variation in the Human Germline. Annu. Rev. Genomics Hum. Genet. 2014;15:47–70.

9. Makova KD, Hardison RC. The effects of chromatin organization on variation in mutation rates in the genome. Nat. Rev. Genet. 2015;16:213–23.

10. Francalacci P, Sanna D, Useli A, Berutti R, Barbato M, Whalen MB, et al. Detection of phylogenetically informative polymorphisms in the entire euchromatic portion of human Y chromosome from a Sardinian sample. BMC Res. Notes. Springer Science + Business Media; 2015;8:174.

11. Poznik GD, Xue Y, Mendez FL, Willems TF, Massaia A, Wilson Sayres MA, et al. Punctuated bursts in human male demography inferred from 1,244 worldwide Y-chromosome sequences. Nat. Genet. 2016;48:593–9.

12. Mendez FL, Poznik GD, Castellano S, Bustamante CD. The Divergence of Neandertal and Modern Human Y Chromosomes. Am. J. Hum. Genet. Elsevier BV; 2016;98:728–734.

13. Francalacci P, Morelli L, Angius A, Berutti R, Reinier F, Atzeni R, et al. Low-Pass DNA Sequencing of 1200 Sardinians Reconstructs European Y-Chromosome Phylogeny. Science. American Association for the Advancement of Science (AAAS); 2013;341:565–9.

14. Hallast P, Batini C, Zadik D, Maisano Delser P, Wetton JH, Arroyo-Pardo E, et al. The Y-Chromosome Tree Bursts into Leaf: 13,000 High-Confidence SNPs Covering the Majority of Known Clades. Mol. Biol. Evol. Oxford University Press (OUP); 2015;32:661–73.
